# Preparation and Evaluation of Liposomes and Niosomes Containing Total Ginsenosides for Anti-Photoaging Therapy

**DOI:** 10.3389/fbioe.2022.874827

**Published:** 2022-04-06

**Authors:** Yuanyuan Jin, Da Liu, Zhen Lu, Lubing Yang, Jiangli Chen, Xuyan Zhou, Zhidong Qiu, Ye Jin

**Affiliations:** ^1^ School of Pharmacy, Changchun University of Chinese Medicine, Changchun, China; ^2^ School of Environment and Quality Testing, Chongqing Chemical Industry Vocational College, Chongqing, China; ^3^ Department of Pharmacy, The Third Affiliated Hospital of Changchun University of Chinese Medicine, Changchun, China

**Keywords:** liposomes, niosomes, total ginsenosides, transdermal delivery, photoaging

## Abstract

Ginsenosides are the principal bioactive compounds of ginseng. Total ginsenosides (GS) contain a variety of saponin monomers, which have potent anti-photoaging activity and improve the skin barrier function. To enhance the efficiency of GS transdermal absorption, GS liposomes (GSLs) and GS niosomes (GSNs) were formulated as delivery vehicles. Based on the clarified and optimized formulation process, GSL and GSN were prepared. The structure, cumulative transmittance, skin retention, total transmittance, and bioactivity of GSLs and GSNs were characterized. GSL and GSN were shown to inhibit lipid peroxidation and increase the contents of superoxide dismutase (SOD) and glutathione peroxidase (GSH-Px) in human keratinocytes (HaCaTs). In addition, HaCAT cell migration, proliferation, and GS cellular uptake were significantly increased. The therapeutic effects of GSL and GSN were also evaluated in a rat model of photoaging. Histopathological changes were assessed in rat skin treated with GSL, GSN, or GS by hematoxylin–eosin (H&E) and aldehyde fuchsine staining. Malondialdehyde (MDA), SOD, GSH-Px, matrix metalloproteinases (MMPs), interleukin-6 (IL-6), interleukin-1β (IL-1β), and tumor necrosis factor-α (TNF-α) expression levels were determined. Results indicated that the optimal formulation of GSL used soybean lecithin (SPC) as the phospholipid, with a lipid–drug ratio of 1:0.4 and a phospholipid–cholesterol ratio of 1:3.5. The optimal temperature for the preparation process of GSN by ethanol injection was 65°C, with a ratio of the organic phase to aqueous phase of 1:9. It was demonstrated that the cumulative release rate, skin retention rate, and total transmission rate of GSL-7 at 24 h were higher than those of GSN-4 and GS. GSL-7 significantly inhibited skin lipid peroxidation caused by ultraviolet (UV) radiation. In addition, GSL-7 reduced the contents of MMPs and inflammatory cytokines in skin tissue. In conclusion, GSL-7 may reduce skin aging caused by UV radiation and contribute to skin tissue repair.

## Introduction

As the body’s first line of defense, the skin protects the body from external damage (Shin et al., 2021). There are generally two forms of skin aging: natural aging and photoaging (Bhattacharya et al., 2019). UV radiation is a major factor in the acceleration of both forms of skin aging by causing excessive reactive oxygen species (ROS) production (Chung 2003; Ho et al., 2005; Wolf et al., 2020). ROS can upregulate heterodimer-activated protein 1 (AP-1), which is composed of c-Fos and c-Jun proteins, by activating mitogen-activated protein kinase (MAPK), which induces the synthesis of matrix-degrading enzymes such as matrix metalloproteinase 1 (MMP-1) and matrix metalloproteinase 3 (MMP-3) (Lee et al., 2020; Mu et al., 2021; Wan et al., 2021). MMPs degrade collagen and elastin in the extracellular matrix *in vivo*. MMP-1 breaks down intact type I and III fibrillar collagen, ultimately leading to progressive senescence of fibroblasts (Rui-Zhen et al., 2004; Zhang and Tian, 2007; Zhou et al., 2021). Additionally, MMP-1 upregulates the expression of IL-6, IL-1β, and TNF-α by activating nuclear factor-κB (NF-κB), which can lead to the inflammatory infiltration of neutrophils and further production of ROS (Xiao et al., 2016; Ighodaro and Akinloye, 2018). Numerous studies have shown that natural antioxidants such as pinus densiflora (Jung et al., 2014), grape seed (Filip et al., 2013), and hawthorn (Liu S. et al., 2018) can inhibit UV radiation-induced skin photoaging by blocking MAPK and NF-κB signaling (Lu et al., 2017).

GS are the main pharmacologically active components of ginseng. More than 40 saponin monomers have been identified, of which Rb1, Rb2, Rb3, Rk3, Rg1, Re, and Rf account for approximately 70% of the GS (Ratan et al., 2021). GS have been shown to repair UV-induced skin cell damage (Jiao et al., 2021). The predominant cause of DNA damage caused by UV radiation is the formation of cyclobutane pyrimidine dimer (CPD) (Liu et al., 2021). GS significantly reduce the expression of CPD by inducing damage-specific DNA-binding protein 2 (Um et al., 2017). In addition, GS inhibit the apoptosis of damaged cells and subsequently upregulate the cellular repair cycle by modulating the expression of JNK and p53 (Bai et al., 2018). UV radiation also increases the expression of ROS and NO in skin cells, leading to DNA strand breaks, purine or pyrimidine oxidation, and lipid peroxidation (Yong et al., 2008; Salama et al., 2018). GS reduce UV-induced ROS and NO production. In addition, they may protect keratinocytes from UVB damage (Park et al., 2009). Oh et al. (2014); Oh et al. (2015); Oh et al. (2016); and Yang et al. (2020) demonstrated that GS Rb1, Rb3, and Rh2 inhibit the expression of UV-induced ROS and MMP expression. GS Rk3 may also decrease MDA expression and increase SOD and GSH-Px expression. Taken together, these results suggest that GS may have anti-photoaging properties in the skin (Nah, 2014; Zhang et al., 2014; Wan et al., 2021). However, the development and utilization of GS is limited by their low bioavailability, poor lipophilicity, and limited transdermal absorption. The most common methods of improving drug bioavailability include extending the skin surface residence time and increasing skin permeability. Selecting the appropriate dosage form may also present an effective approach to overcome these issues (Xu et al., 2003; Kang et al., 2009).

Liposomes are microvesicles composed of one or more lipid bilayers that can be used as drug delivery systems (Mao et al., 2015). Hydrophilic drugs can be embedded in the hydrophilic central region, and lipophilic drugs can be embedded in the lipid bilayers (Raj et al., 2018). Liposomes can be used for the targeted and controlled release of drugs (Rideau et al., 2018). The membrane structure of liposomes is biocompatible with the lipids in the cuticle of the skin, meaning that liposomes can enter cells or adsorb to the outer layer of target cells (Bozzuto and Molinari, 2015). Liposomes can also be metabolized and degraded by the body’s metabolic enzymes, thereby reducing the risk of adverse drug reactions (Rideau et al., 2018). Thus, liposomes are often used as carriers for transdermal drug delivery to increase solubility, prevent degradation by enzymes, and prolong the release time (Gautam and Prajapati, 2012; Ahmed et al., 2019; Lu et al., 2020). Niosomes are closed bilayer structures composed of non-ionic surfactants (Gomaa et al., 2014; Vitorino et al., 2014). They have a biofilm structure similar to liposomes (Gurrapu et al., 2012; Moghassemi et al., 2015; Seleci et al., 2016; Chen et al., 2019). Because of the presence of non-ionic surfactants, niosomes have a higher stability than liposomes when stored (Abaee and Madadlou, 2016; Bi et al., 2019). Non-ionic surfactants can also enhance the transdermal absorption efficiency of drugs (Jacob et al., 2017; Barani et al., 2020; Farmoudeh et al., 2020).

To explore the application of these vehicles for GS delivery, GS were embedded in liposomes and niosomes. The effects of liposomes and niosomes on the percutaneous administration of GS were evaluated using an optimized formulation process, measuring the effects of several parameters on transdermal absorption efficiency, such as particle size, ζ-potential, and encapsulation efficiency (EE%). The effects of GSL and GSN on HaCaT cells were investigated by cell proliferation, wound healing, and uptake assays. Furthermore, the therapeutic effects of GSL and GSN on rat skin photoaging were evaluated by determining the expression levels of MDA, SOD, GSH-Px, MMPs, and inflammatory cytokines in rat skin.

## Materials and Methods

### Materials

GS, mannitol, fluorescein isothiocyanate (FITC), octadecylaminetech (ODA), and cholesterol were all purchased from Shanghai Yuanye Biological Co., LTD. (Shanghai, China). SPC, egg phosphatidylcholine (Egg-PC), hydrogenated soybean lecithin (HSPC), and two palmitoyl phosphatidylcholine (DPPC) were obtained from YiWeiTa Pharmaceutical Technology Co., LTD. (Shanghai, China). SOD, GSH-Px, MDA, and the bicinchoninic acid (BCA) kit were all purchased from Nanjing Jiancheng Institute of Biological Engineering, (Nanjing, China). Dicetaceumphosphate (DCP) was from Sigma-Aldrich (Shanghai, China). All enzyme-linked immunosorbent assay (ELISA) kits used in this experiment were from Jiangsu MEIMIAN Co., Ltd. (Jiangsu, China). The animals were purchased from Changchun Yisi Experimental Animal Technology Co., Ltd. (Certificate No.: SCXK (JI) -2019-0003). Other reagents were all of the analytical grade. DMEM was from Gibco (Grand Island, New York, United States). The phosphate-buffered solution (PBS), fetal bovine serum (FBS), and penicillin–streptomycin solution were obtained from Hyclone (Logan, UT, United States). The cell counting kit-8 (CCK-8) was obtained from Wuhan Boshide Biological Engineering Co. Ltd. (Wuhan, China). The HaCaT cell was from BeNa Culture Collection (Suzhou, China).

### Preparation of GSL and GSN

The GSL was prepared by the ethanol injection method. Firstly, phospholipids, GS, and cholesterol were dissolved in anhydrous ethanol at a certain molar ration to form a mixture. Then, the lipid solution was slowly injected into the PBS under stirring at a volume ratio of 1/9. GSL suspension was thus obtained and anhydrous ethanol was removed by rotary evaporation. In these experiments, the formulation of GSLs was optimized by changing different prepared parameters including phospholipid species, lipid-drug ratio, and phospholipid to cholesterol ratio. The optimized parameters of GSL are shown in Table 1. To improve stability, the GSL suspension was freeze-dried to obtain GSL freeze-dried powder. 0.1 g of lyophilized protectant (mannitol) was added to 10 ml of GSL. After mixing, it was pre-frozen at −80°C and freeze-dried for 48 h to obtain GSL freeze-dried powder.

**TABLE 1:** Formulation optimization of GSL.

Sample	Phospholipid species	Lipid–drug ratio (n/n)	Phospholipid–cholesterol ratio (n/n)
GSL-1	SPC	1/0.2	1/2.5
GSL-2	Egg-PC	1/0.2	1/2.5
GSL-3	HSPC	1/0.2	1/2.5
GSL-4	DPPC	1/0.2	1/2.5
GSL-5	SPC	1/0.4	1/2.5
GSL-6	SPC	1/0.5	1/2.5
GSL-7	SPC	1/0.4	1/3.5
GSL-8	SPC	1/0.4	1/4.0
GSL-9	SPC	1/0.4	1/4.5

GSL, total ginsenoside liposome.

The preparation method of GSN was similar to that of GSL. Firstly, 79 mg of span-80, 396 mg of tween-80, 190.90 mg of cholesterol, and 10.09 mg of DCP were dissolved in a certain volume of anhydrous ethanol by ultrasonication. Then, the aforementioned mixture was mixed with GS. The GSN suspension was thus obtained and anhydrous ethanol was removed by rotary evaporation. The process parameters for GSN are shown in Table 2. The GSN was prepared by controlling the heating temperature and the organic phase to water phase ratio. The freeze-drying method of GSN freeze-dried powder was similar to GSL freeze-dried powder.

**TABLE 2:** Formulation optimization of GSN.

Sample	Heating temperature (°C)	Organic phase–water phase ratio (%)
GSN-1	60	10
GSN-2	60	15
GSN-3	60	20
GSN-4	65	10
GSN-5	65	15
GSN-6	65	20
GSN-7	70	10
GSN-8	70	15
GSN-9	70	20

GSN, total ginsenoside niosome.

### Characterization of GSL and GSN

In this experiment, the particle size, dispersion, and ζ-potential of GSL and GSN were determined by dynamic light scattering (DLS, Nano-ZS ZEN3600, Malvern, United Kingdom). The structure of GSL and GSN was measured by using a transmission electron microscope (TEM) at a 3-kV accelerating voltage (Japan, HITACHI H-7650). A differential scanning calorimeter (DSC, Germany, METTLER TOLEDO, DSC3) was used to evaluate the interaction between the total ginsenosides and membrane materials.

In addition to these, the concentration of GS was determined by high-performance liquid chromatography (HPLC, America, Agilent, LC-1260). The EE% of both GSL and GSN was determined by an ultracentrifugation method. The freshly prepared GSL-7 and GSN-4 were centrifuged at 4,000 rpm for 30 min and the supernatant was separated from the particles. The latter, when subtracted from the GS amount, gave the entrapped GS. The absorption wavelength of GS is 203 nm. 1.3 ml/min of acetonitrile and 0.1% of phosphate water were used as the mobile phase. The EE% of GSL and GSN were evaluated by HPLC and calculated by the following equation:

EE (%)=1−W1/W2×100%, (1)

where the content of free GS is W1 and the total content of GS in GSL or GSN is W2.

### Transdermal Absorption of GSL and GSN

In this study, the Franz diffusion pool method was used to evaluate the transdermal absorption (Bi et al., 2019). The animal experiments were approved by the Institutional Animal Care and Use Committee of Changchun University of Chinese Medicine, the animal ethical review number is 20190130. SD rats were fed freely for 24 h according to clean standards. Before the transdermal absorption experiment, the rats were euthanized with pentobarbital sodium. A piece of rat abdomen skin was harvested without hair, then after removing the skin moisture with filter paper, the skin was placed between a receiving pool and supply pool, and then PBS (pH 7.2) solution was used as the receiving medium and placed in the receiving pool. One ml of GS, GSL, and GSN solution with the same content of GS (200 mg) was added into the supply pool. The temperature was controlled at 37°C and the speed was set as 300 r/min. Then, 1 ml of GS, GSL, or GSN solution was removed from the receiving pools at 6, 8, 12, 24, and 48 h. Then replaced equivalent fresh liquid was added into the receiving pool. The concentration of GS was measured by HPLC.

The residual GS on the skin surface was cleaned, the skin was cut into pieces, and was then homogenized with saline at 800 rpm for 30 min. The supernatant solution was added into 5 ml methanol with a vortex for 30 s. The aforementioned solution was processed by a 0.22-μm membrane. Finally, the transdermal absorption efficiency of GS, GSL, or GSN was calculated by the following equation:

$$\text{Q}=\left[\overset{\text{n}-1}{\underset{\text{i}=1}{\sum }}\text{Ci ∗ V}+\text{Cn ∗ V}0\right]/\text{S,}$$ (2)

where Q: the transdermal absorption efficiency of GS. Cn: concentration of GS at the nth hour. Ci: concentration of GS at the ith hour. V0: volume of the accept pool. V: volume of the sampling. S: diffusion area (2.23 cm^2^).

### The Proliferation and Uptake of GSL and GSN on HaCaT Cells

HaCaT cells were cultured in DMEM supplemented with 10% FBS and 1% penicillin–streptomycin solution at 5% CO_2_ and 37°C.

#### Cell Viability

The cell viability was measured by a CCK-8 assay. Briefly, HaCaT cells (5 × 10^3^ cells/well) were cultured in 96-well plates and incubated for 24 h at 37°C. After that, different concentrations of the GS (0, 5, 10, 20, 40, 60, 80, 100, and 200 μg/ml) were added into each well for another 24-h incubation. Ten μl of the CCK-8 solution was added to each well at 10% dilution. The cells were then incubated for 1 h, and the mean optical density (OD) was measured at 450 nm using a microplate reader. All experiments were performed in triplicate. The percentage of cell viability was calculated according to the following formula:

$$\text{Percentage of cell viability}=\;\frac{\text{OD treatment group}}{\text{OD control group}}\times 100\text{\%}\text{.}$$ (3)

#### GS, GSL-7, and GSN-4 on the Oxidation of HaCaT Cells

HaCaT cells with a density of 5 × 10^3^ cells/well were inoculated in 96-well plates and cultured overnight. Then, different concentrations (10, 20, and 40 μg/ml) of GS or GSL-7 and GSN-4 with a drug loading of 20 μg/ml were added in 96-well plates and incubated for 24 h. Subsequently, the solution in the 96-well plates was removed, and the cells were washed three times with PBS. After that, the MDA, SOD, and GSH-Px were tested by a plate reader (SER33270-1236, Molecular Devices, United States).

#### Cellular Uptake Quantitative Analysis

##### Preparation of GSL-7-FITC and GSN-4-FITC

GSL-7 and GSN-4 were stained with FITC to study the HaCaT cell uptake. Firstly, 20 mg of ODA and 28 mg of FITC were dissolved in 6 ml of anhydrous ethanol and stirred in the dark for 24 h. Then, 10 times the amount of water was added, filtered through a microporous membrane, and dried at room temperature in the dark to obtain ODA-FITC. GSL-7-FITC was prepared by the ethanol injection method. Firstly, phospholipids, GS, cholesterol, and 10 mg of ODA-FITC were dissolved in anhydrous ethanol at a certain molar ration to form a mixture. Then, the lipid solution was slowly injected into PBS (pH 7.4) under stirring at a volume ratio of 1/9. GSL-7-FITC was thus obtained and the anhydrous ethanol was removed by rotary evaporation. Free ODA-FITC was removed by ultrafiltration. The preparation process needs to be protected from light throughout the process. GSN-4-FITC was prepared by the same method.

##### Cellular Uptake of GSL-7 and GSN-4

The HaCaT cells (5 × 10^4^ cells per well) were seeded in confocal dishes and incubated for 24 h. The medium solution containing GSL-7, GSN-4, GSL-7-FITC, and GSN-4-FITC (the concentration of ODA-FITC in each group was 1 μg/ml) was added and incubated for 1 h, washed with PBS three times, added 400 μl DAPI dye solution, incubated at 37°C for 10 min, discarded the solution, washed three times with PBS, and then observed and recorded under a confocal microscope (ZEISS, Germany).

The HaCaT cells were seeded in six-well plates at a density of 2 × 10^5^ cells per well and incubated for 24 h. The medium solution containing GSL-7, GSN-4, GSL-7-FITC, and GSN-4-FITC (the concentration of ODA-FITC in each group was 1 μg/ml) was added and incubated for 1 h and washed with PBS three times. After trypsinization, the cells were resuspended in PBS, and the fluorescence intensity of FITC in each group was detected by flow cytometry (Beckman Cytoflex, United States).

#### Wound Healing and Cell Proliferation Assay

The HaCaT cells were seeded in a six-well plate at a density of 5 × 10^5^ cells/well and were adhered overnight. Subsequently, a 10-μl pipette tip was utilized to produce uniform scratches. Then, serum-free DMEM was added with the same concentration (20 μg/ml) of GS, GSL-7, and GSN-4, and incubated with the cells for another 24 h. The migration profile was recorded by using an inverted fluorescence microscope and the widths of the scratches were measured by the ImageJ software (NIH, United States).

$$\text{Healing rate\%}=\frac{0\text{ h wound area}-24\text{ h wound area}}{0\text{ h wound area}}\times 100\text{\%}\text{.}$$ (4)

#### Proliferation of HaCaT Cells

The HaCaT cells were inoculated in a 12-well plate at a density of 5 × 10^4^ cells/well and cultured overnight. Then, the same concentration (20 μg/ml) of GS, GSL, and GSN was added, and incubated with the cells for another five days. Every other day count and a microscope (EVOS, United States) were used to record the images.

### GSL and GSN for Repairing Photoaging

Skin photoaging refers to long-term skin damage caused by UV radiation. GS effectively improve the situation. GS liposomes and niosomes were prepared to enhance the skin retention and improve the curative effect. Firstly, SD female rats were divided into five groups and each group included eight rats. The groups were recorded as follows: control group (shaving, no irradiation and treatment), model group (shaving and irradiation, no treatment), GS group, GSL group, and GSN group (GS, GSL, and GSN: shaving, irradiation, and treatment).

Before the experiment, the rats were firstly fed normally for seven days and fed freely for 24 h. Then, the back hair of the rats was shaved and the area was about 3 cm × 3 cm. Ultraviolet (UVA + UVB) irradiation was used to irradiate the naked back skin of the rats to establish the photoaging model (Rui-Zhen et al., 2004).

Except for the control group, the shaved rats were put into the self-made UV-lamp box and the fixed irradiation distance was 20 cm for ultraviolet (UVA + UVB) irradiation. The irradiated time was 20 min/day in the first week, 40 min/day in the second week, and 60 min/day in the third week. The photoaging model was successfully established when the skin on the back of the rats was dry, peeling, and even developed local ulceration. After modeling, the other three groups were given the same concentration (500 μg/ml) and the same dosage (0.5 ml/day) of GS, GSL, and GSN for treatment, except the control group and model group. The GS, GSL, or GSN was evenly applied on the skin of the affected area on the back of the rats and the skin of the rats in each group was observed after 10 days of continuous administration (Filip et al., 2013; Liu S. et al., 2018)

Five groups of rats were sacrificed after anesthesia to harvest the exposed target skin, the skin was fixed by 4% paraformaldehyde, and embedded in paraffin. Finally, the skin tissues were stained by the H&E and aldehyde fuchsin staining methods to analyze the histology of epidermis, dermis, collagen fibers, and elastic fibers (Gurrapu et al., 2012).

In addition, the effects of GSL-7 and GSN-4 on MDA, SOD, GSH-Px activities, MMPs content, IL-6, IL-1β, and TNF-α ontent were also measured using an ELISA kit in accordance with the manufacturer’s protocol by a plate reader (SER33270-1236, Molecular Devices, United States).

### Statistical Analysis

Comparisons of two or multiple groups were analyzed by using *t*-tests (Mann–Whitney test), *p* < 0.05 indicated a significant difference and *p* < 0.001 indicated a highly significant difference. The results were expressed as mean ± standard deviation.

## Results and Discussion

### Preparation of GSL and GSN

EE% is a key parameter of GSL and GSN quality. To prepare GSL and GSN with high EE%, the formulation process was optimized by changing the temperature and the ratios of the organic phase to aqueous phase, drug to phospholipid, and phospholipid to cholesterol. It can be observed from Tables 1, 3 that when preparing GSL, the phospholipid species influenced the EE% of GSL. The EE% of GSL was highest when the phospholipid used was SPC. The EE% of GSL was highest at an optimal lipid-drug ratio. When the ratio of phospholipid to cholesterol was increased to 1:3.5, the EE% of GSL was 62.43%. As the amount of lipids affects the drug-loading capacity, drugs beyond the upper limit cannot be loaded. Cholesterol can improve the fluidity of the lipid membrane and improve the EE%, but excessive cholesterol content will lead to the decrease of EE% and drug leakage. Therefore, the optimal parameters for preparing GSL used SPC as the phospholipid, with a lipid-drug ratio of 1:0.4 and a phospholipid to cholesterol ratio of 1:3.5. The sample was recorded as GSL-7. For preparing GSN, it can be seen from Tables 2, 3 that the EE% of GSN increased at first and then decreased with increasing temperature. When the heating temperature was 65°C, the EE% of GSN was the highest (56.50%). Similarly, the EE% of GSN was initially higher and then decreased with an increasing ratio of the organic phase to aqueous phase, which may compete with the drug for packing space within the bilayer and limit the assembly of the drug into the vesicle. Similar results have also been observed in other studies (Gomaa et al., 2014; Vitorino et al., 2014). When the ratio of the organic phase to aqueous phase was 10%, the EE% of GSN was 56.50%. Therefore, the optimal preparation process of GSN was as follows: the temperature was 65°C and the ratio of the organic phase to aqueous phase was 10%. The sample was recorded as GSN-4.

**TABLE 3:** Encapsulation efficiency of GSL and GSN.

Sample	Encapsulation efficiency (%)	Sample	Encapsulation efficiency (%)
GSL-1	45.50 ± 1.07	GSN-1	28.79 ± 1.50
GSL-2	40.74 ± 1.10	GSN-2	39.14 ± 1.58
GSL-3	36.89 ± 1.23	GSN-3	50.89 ± 0.40
GSL-4	34.90 ± 1.53	GSN-4	56.50 ± 0.66
GSL-5	55.45 ± 0.93	GSN-5	50.56 ± 0.92
GSL-6	51.32 ± 1.54	GSN-6	56.52 ± 0.78
GSL-7	62.43 ± 2.25	GSN-7	43.52 ± 1.05
GSL-8	53.45 ± 1.12	GSN-8	34.10 ± 1.13
GSL-9	45.95 ± 0.45	GSN-9	52.86 ± 0.30

### Characterization of GSL-7 and GSN-4

#### Morphology, Particle Size, and ξ-potential of GSL-7 and GSN-4

**FIGURE 1:**
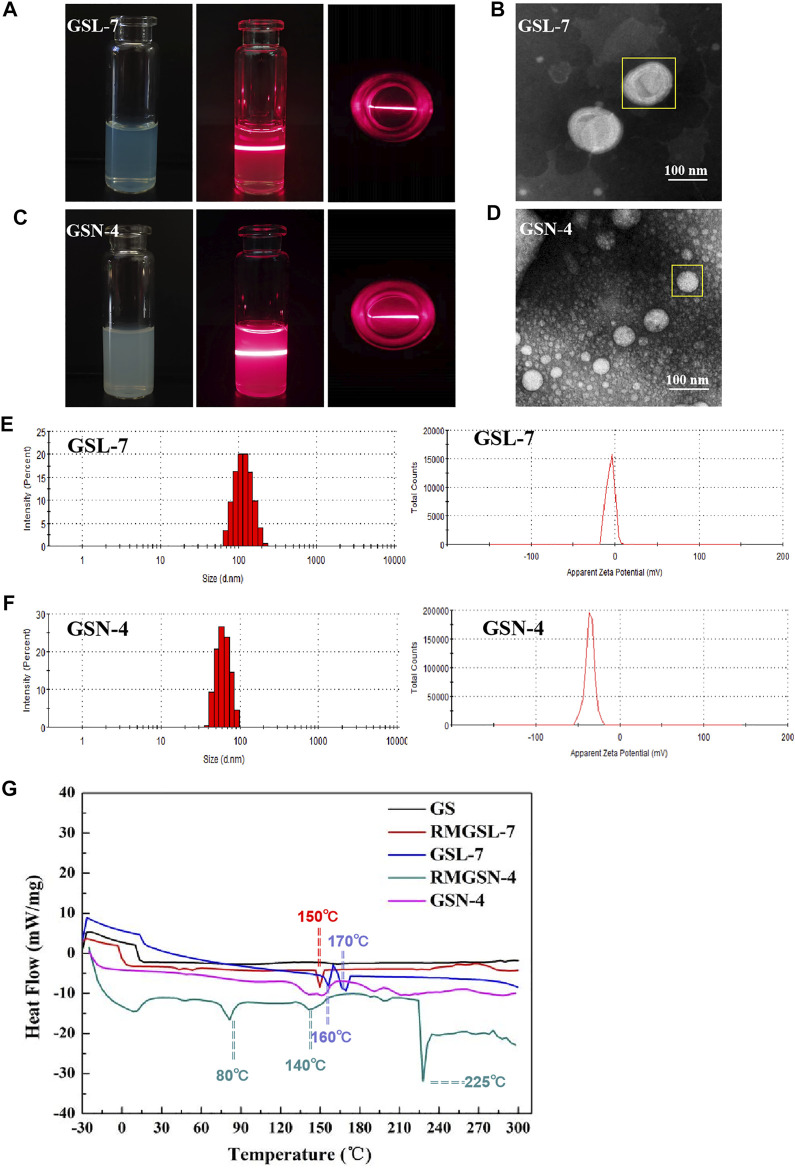
Characterization evaluation of GSL-7and GSN-4. **(A)** Appearance and Tyndall effect of GSL-7; **(B)** TEM images of GSL-7. Scale bar: 100 nm; **(C)** Appearance and Tyndall Effect of GSN-4; **(D)** TEM images of GSN-4. Scale bar: 100 nm; **(E)** Size distribution and ξ-potential of GSL-7; **(F)** Size distribution and ξ-potential of GSN-4. **(G)** DSC curves of GSL-7 and GSN-4. Data are presented as *N* = 3. Abbreviations: GSL-7, total ginsenosides liposomes; GSN-4, total ginsenosides niosomes; RMGSL-7, a raw material mixture of prepared total ginsenosides liposomes; RMGSN-4, a raw material mixture of prepared total ginsenosides niosomes.

As shown in Figures 1A, C, the appearance of GSL-7 and GSN-4 was a clear and uniform liquid. When a beam of infrared light was passed through GSL-7 and GSN-4, the Tyndall effect was observed, indicating that GSL-7 and GSN-4 were polymer colloidal solutions (Liu Q. et al., 2018). The microstructure of GSL-7 and GSN-4 was determined by TEM. GSL-7 and GSN-4 both exhibited spherical shapes and uniform sizes (Figures 1B, D). The diameter of GSL-7 was ∼100.0 nm and the diameter of GSN-4 was ∼60.0 nm.

The particle size and ξ-potential of GSL-7 and GSN-4 were determined by DLS (Figures 1E,F). The particle size of GSL-7 was 110.0 ± 3.5 nm, the polydispersity index (PDI) was 0.056 ± 0.009, and the ξ-potential was −5.7 ± 0.5 mV. The particle size of GSN-4 was 60.93 ± 0.17 nm, the PDI was 0.086 ± 0.016, and the ξ-potential was −35.5 ± 1.8 mV. The particle size was consistent with the TEM data, and GSL had a larger particle size and a more uniform particle-size distribution than GSN-4.

#### Differential Scanning Calorimeter (DSC) of GSL-7 and GSN-4

DSC analyses were performed to determine whether the GSL-7 or GSN-4 could be successfully prepared, and to understand the nature and physical state of the GS, liposomes, and niosomes (Figure 1G). Compared with GS and the raw material mixture of GSL-7 (RMGSL-7), there was a marked endothermic peak shift at 150°C and the emergence of a new endothermic peak at 160 and 170°C for GSL-7. It can be inferred that GS and the raw materials of the prepared GSL-7 are not simply physically mixed, but interact, and that GS is embedded in the double-layer structure of phospholipids. Similarly, the raw material mixture of GSN-4 (RMGSN-4) had noticeable endothermic peaks at 80, 140, and 225°C, indicating that there was an interaction between GS and the raw materials. These endothermic peaks were not present in the DSC curves of GSN-4, demonstrating that a chemical interaction had not occurred between the GS and the niosomal system, and the GSN-4 was successfully prepared.

### Transdermal Absorption of GSL-7 and GSN-4

The cumulative transmittance, skin retention rate, and total transmittance of GS, GSL-7, and GSN-4 were measured. The results are shown in Table 4. It can be observed that a minimal amount of GS was detected within 48 h. The cumulative release rate, skin retention rate, and total penetration rate of GSL-7 at 48 h were higher than those of GSN-4, at 6.43, 10.42, and 16.85%, respectively. Both GSL-7 and GSN-4 improved the skin penetration rate of GS, but the effects of GSL-7 were more pronounced, with more than 85% of GS penetrating the skin barrier within 24 h. GSL-7 tended to be absorbed by the skin, which might be because of the structure and carrier material of GSL-7. These properties may improve the effect of GSL-7 on skin anti-photoaging.

**TABLE 4:** Cumulative transmittance, retention, and total transmittance of GS in the skin.

Time (h)	Accumulative release rate (%)			Skin retention rate (%)			Total skin transmittance (%)		
	GS	GSL-7	GSN-4	GS	GSL-7	GSN-4	GS	GSL-7	GSN-4
6	0.12 ± 0.01	21.43 ± 0.91	9.05 ± 1.83	0.46 ± 0.07	26.74 ± 2.68	16.78 ± 0.06	0.58 ± 0.06	48.17 ± 2.08	25.83 ± 2.40
8	0.19 ± 0.01	23.52 ± 2.33	11.31 ± 0.93	0.57 ± 0.01	30.92 ± 1.04	18.37 ± 0.50	0.76 ± 0.01	54.44 ± 1.34	29.68 ± 0.48
12	0.35 ± 0.02	29.40 ± 1.69	14.13 ± 0.79	0.85 ± 0.01	44.49 ± 0.95	31.98 ± 1.97	1.20 ± 0.01	73.89 ± 2.36	46.11 ± 1.17
24	0.72 ± 0.03	33.30 ± 2.29	26.15 ± 1.03	0.99 ± 0.01	52.16 ± 1.35	42.67 ± 1.90	1.72 ± 0.03	85.46 ± 3.64	68.82 ± 0.86
48	0.79 ± 0.05	34.21 ± 1.80	27.78 ± 0.89	1.02 ± 0.05	53.63 ± 0.49	43.21 ± 1.29	1.81 ± 0.05	87.84 ± 1.77	70.99 ± 0.39

GSL-7, total ginsenoside liposome-7; GSN-4, total ginsenoside niosome-4.

### GSL and GSN for the Proliferation and Uptake of HaCaT Cells

#### GS, GSL, and GSN Enhanced the Viability and Antioxidative Potential of HaCaT Cells

**FIGURE 2:**
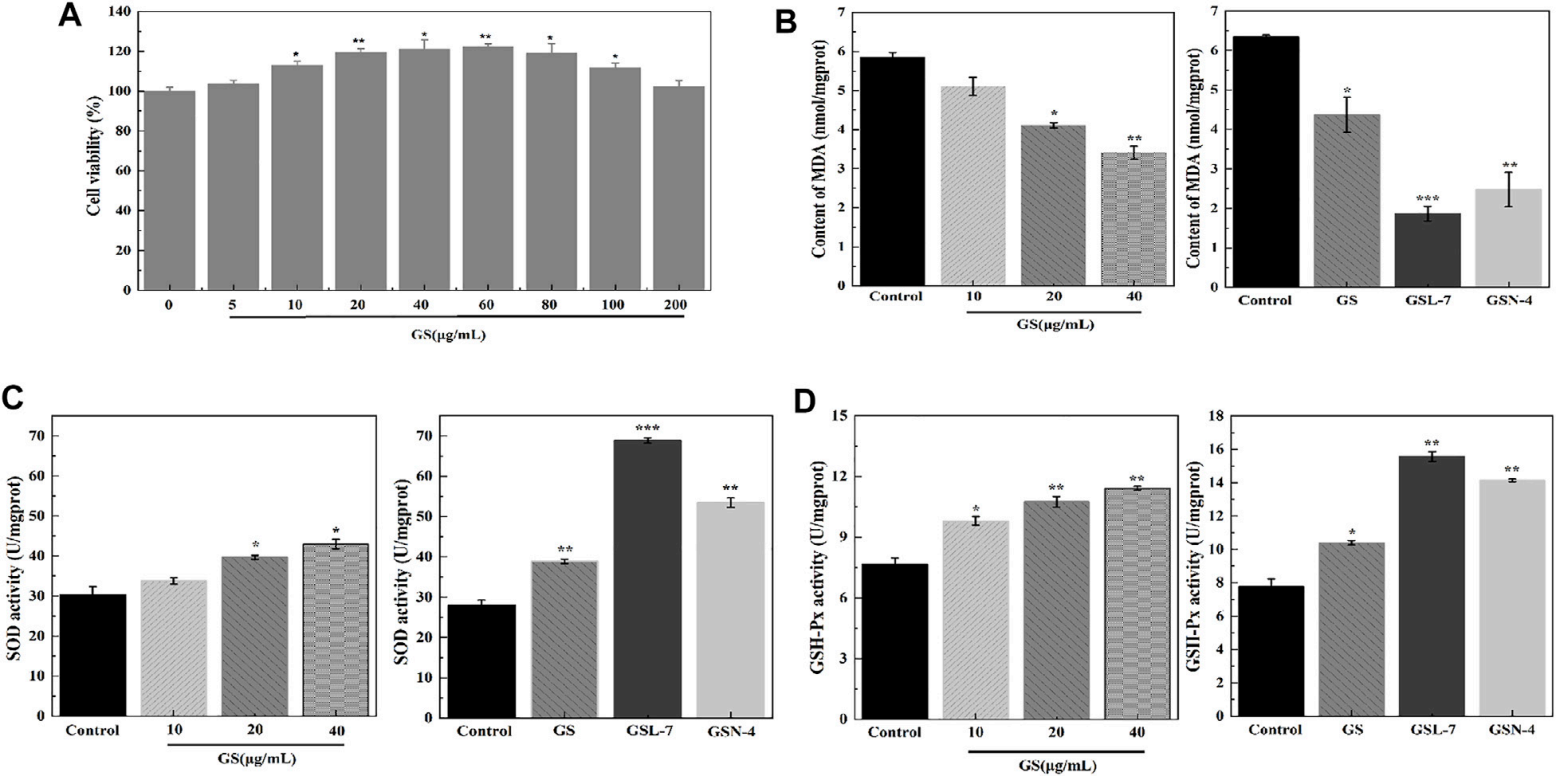
GS, GSL, and GSN enhanced viability and anti-oxidative effects of HaCaT cells. **(A)** CCK-8 assay of GS (*n* = 3). **(B)** Effects of GS, GSL, and GSN on MDA content of HaCaT cells. **(C)** Effects of GS, GSL, and GSN on SOD content of HaCaT cells. **(D)** Effects of GS, GSL, and GSN on GSH-Px content of HaCaT cells. ***p < 0.001, GSL-7 group or GSN-4 vs control group, **p < 0.01, GSL-7 group or GSN-4 vs control group, *p < 0.05, GSL-7 group or GSN-4 group vs control group; Data are presented as *N* = 3, * represent significant difference between control group and treatment group. Abbreviations: GSL-7, total ginsenoside liposomes; GSN-4, total ginsenoside niosomes.

The concentration of GS was determined to evaluate the biocompatibility of GS. The analysis results show that the cell viability of the GS group was significantly higher than that of the control group (Figure 2A). As GS concentration increased, the cell viability initially increased before decreasing. The cell viability reached 119.57 ± 1.72%, 121.23 ± 4.51%, and 122.47 ± 1.34% with GS concentrations of 20, 40, and 60 μg/ml, respectively, indicating that GS had good biosafety. Therefore, 20, 40, and 60 μg/ml were determined as the administered concentrations for subsequent cell experiments.

To further understand the molecular mechanisms associated with the beneficial effects of GS, oxidative stress levels and endogenous antioxidant enzyme activity were measured in the HaCaT cells. MDA, SOD, and GSH-Px activity was determined using ELISA kits. Compared with the control group, the MDA expression was decreased at all GS doses (20, 40, 60 μg/ml). At the 20 μg/ml GS dose, the MDA content was significantly decreased in the GSL (***p < 0.001) and GSN (**p < 0.01) groups compared with the controls. SOD and GSH-Px activities were significantly increased in the GSL (***p < 0.001) and GSN (**p < 0.01) groups compared with the controls, particularly in the GSL-7 group.

During cell growth, intrinsic antioxidant systems emerge to reduce the levels of ROS and improve cell survival (Ighodaro and Akinloye, 2018). Among them, GSH-Px is known to catalyze the reduction of hydrogen peroxide and other peroxides, while SOD can catalytically reduce O_2_ to hydrogen peroxide. The results of the present study showed that GS, GSL-7, and GSN-4 upregulated SOD and GSH expression, and downregulated the levels of MDA *in vivo*.

#### Cellular Uptake Quantitative Analysis

**FIGURE 3:**
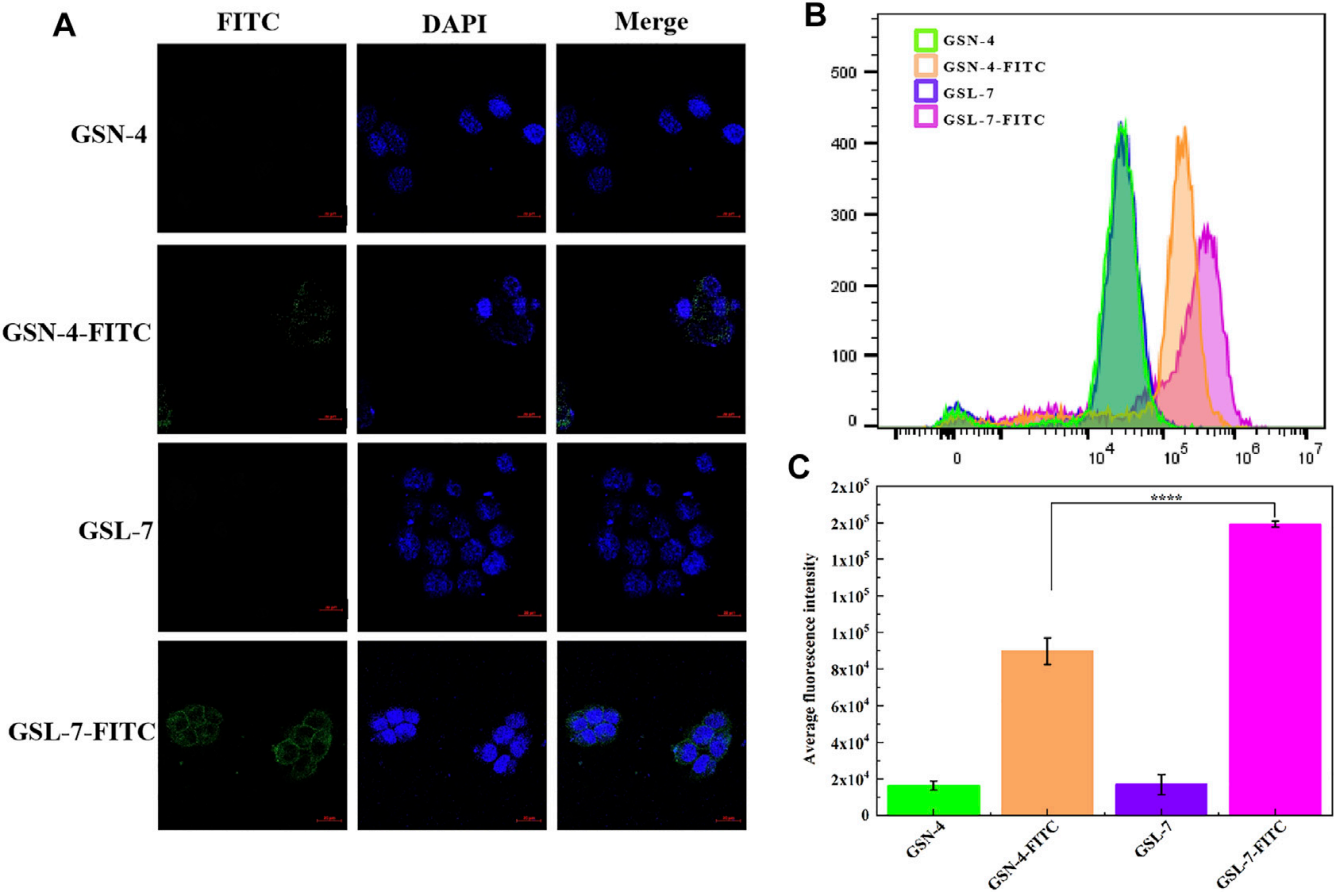
*In vitro* evaluation of GS, GSL, and GSN in the promotion of cellular uptake. **(A)** HaCaT cell marker immunofluorescence staining by confocal laser scanning microscopy. Scale bar: 20 μm **(B)** Detection of FITC fluorescence intensity in different administration groups by flow cytometry **(C)** Quantitative analysis (*n* = 3). ****p ≤ 0.0001, GSL-7-FITC group vs GSN-4-FITC group, * represents significant difference between control group and treatment group. Data are presented as *N* = 3. Abbreviations: GSL-7, total ginsenosides liposomes; GSN-4, total ginsenosides niosomes; GSL-7-FITC, GSL-7 link with FITC fluorescent markers; GSN-4-FITC, GSN-4 link with FITC fluorescent markers.

Cellular uptake is a key step in facilitating intracellular delivery. An analysis showed that GSL-7 and GSN-4 had no self-luminescence, whereas GSL-7-FITC and GSN-4-FITC had green fluorescence (Figure 3A). Compared with GSN-4-FITC, the GSL-7-FITC group had stronger fluorescence. The uptake of the GSL-7-FITC group was quantitatively analyzed by flow cytometry, as shown in Figure 3B. The results were consistent with those observed under the fluorescent microscope. The fluorescent intensity of the GSL-7-FITC group was 2.2-folder higher than the GSN-4-FITC group (Figure 3C), indicating that the liposome structure may aid HaCaT cell uptake.

#### Proliferation of HaCaT Cells

**FIGURE 4:**
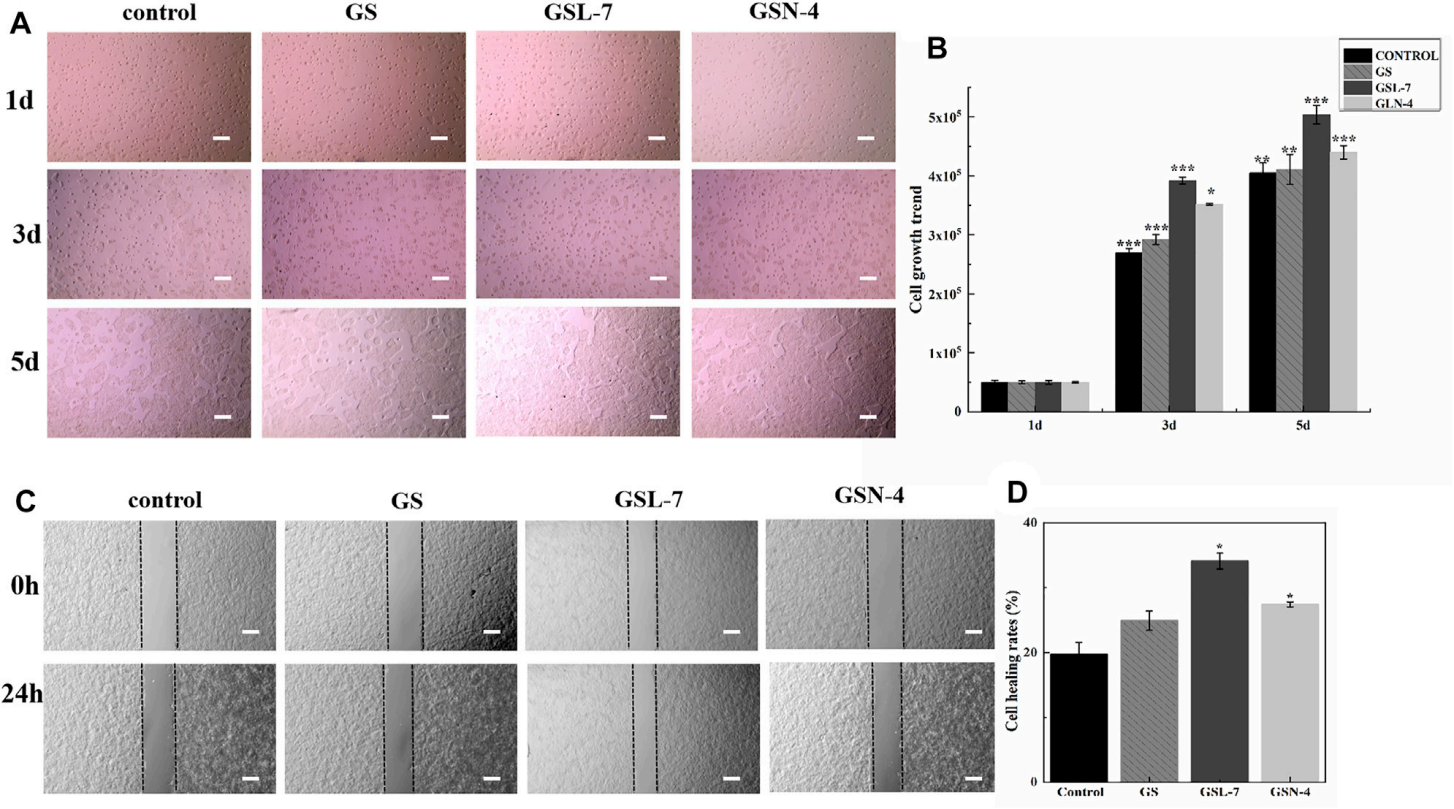
*In vitro* evaluation of GS, GSL-7, and GSN-4 in the proliferation of HaCaT cells. **(A)** HaCaT cell growth diagram. Scale bar: 200 μm. **(B)** HaCaT cell growth trend. **(C)** GS, GSL-7, and GSN-4 promotion the migration of HaCaT cells by wounding healing assay. Scale bar: 200 μm. **(D)** Quantitative analysis. *p < 0.05, **p ≤ 0.01, ***p ≤ 0.001, GSL-7 group or GSN-4 group vs control group. * represents significant difference between control group and treatment group. Data are presented as *N* = 3. Abbreviations: GSL-7, total ginsenoside liposomes; GSN-4, total ginsenoside niosomes.

To investigate whether GS, GSL-7, and GSN-4 affected the proliferation of HaCaT cells, differences in cell growth between the GS, GSL-7, and GSN-4 groups and controls were analyzed. The HaCaT cells were counted every other day and recorded by a microscope. The results are shown in Figure 4A. On the first day, the cells in each group began to anchor to the plate surface. On the third day, cell proliferation of the GS, GSL-7, and GSN-4 groups was noticeably higher than the control group. A quantitative analysis indicated that compared with the 5 × 10^4^ HaCaT cells present at the time of inoculation, cell proliferation increased 5-fold, 5.8-fold, 7.8-fold, and 7.0-fold in the control, GS, GSL-7, and GSN-4 groups, respectively. On the fifth day, cell proliferation was higher in the GSL-7 and GSN-7 groups compared with the GS and control groups; the cell proliferation increased 8-fold, 8.2-fold, 10.0-fold, and 8.8-fold in the control, GS, GSL-7, and GSN-4 groups, respectively. These trends suggest that liposomes and niosomes may enhance the proliferative effect of GS on HaCaT cells. The proliferation trend of the cells in the control group was in line with previously reported results (Wilson 2014).

A wounding healing assay was performed to assess the influence of the GS, GSL, and GSN on the migration properties of HaCaT cells *in vitro*. Changes in the scratch width of each group were observed for 24 h to assess HaCaT-cell migration (Figures 4C,D). GS, GSL-7, and GSN-4 all promoted the HaCaT-cell migration. However, GSL-7 induced the surrounding HaCaT cells to crawl toward the center of the scratch, almost filling the gap within 24 h, showing the smallest scratch width in all treatment groups (GS, GSL-7, and GSN-4 groups) (Figure 4C) with a healing rate of 34.12 ± 1.22% (*p* < 0.05) (Figure 4D).

### Repair of Photoaging by GSL-7 and GSN-4

#### Effects of GSL-7 and GSN-4 on MDA, SOD, and GSH-Px Expression

**FIGURE 5:**
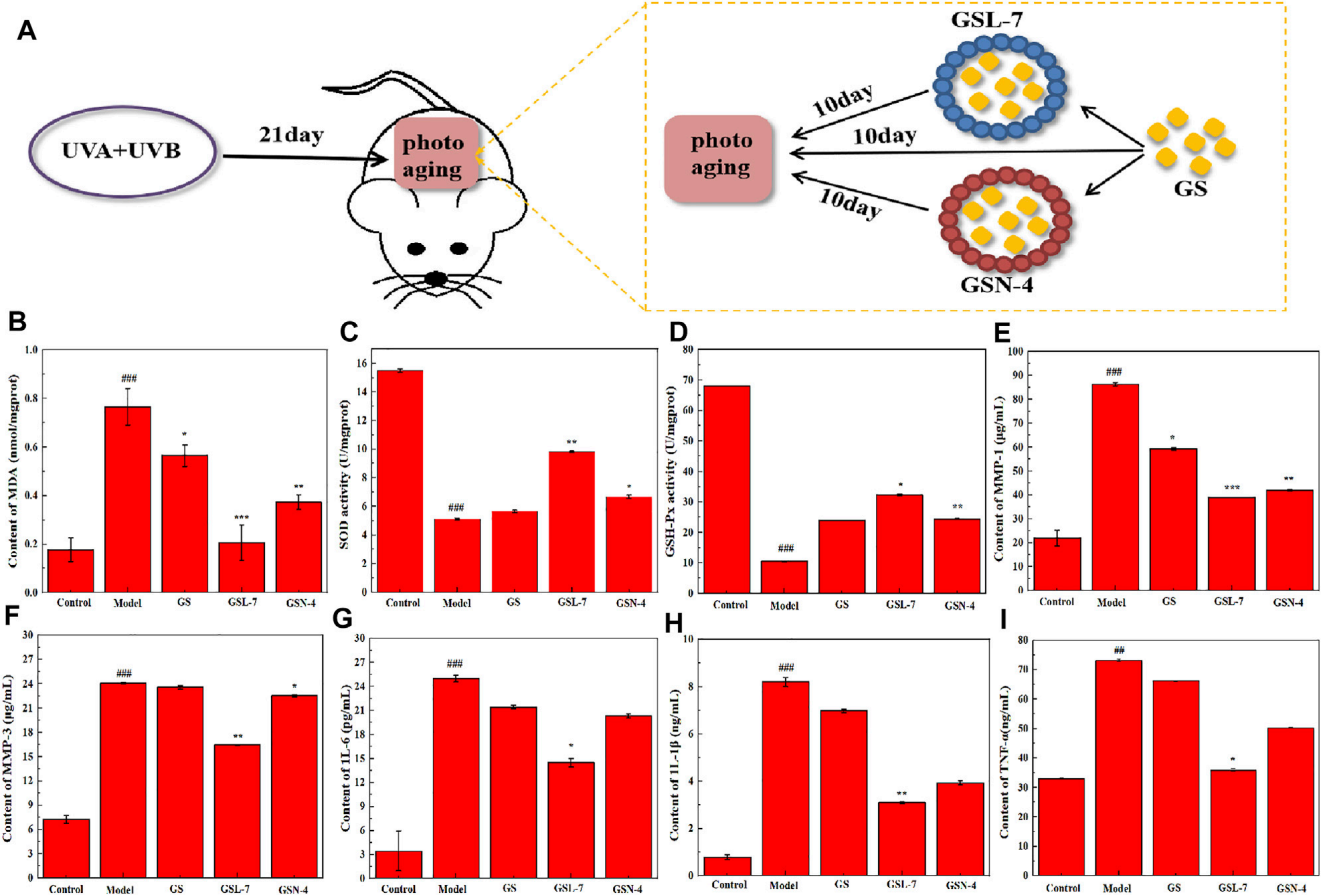
Biological effects of GSL and GSN on the skin. **(A)** Schematic representations of repair photoaging. **(B)** Effects of GSL-7 and GSN-4 on MDA content in rat skin tissues. **(C)** Effects of GSL-7 and GSN-4 on SOD content in rat skin tissues. **(D)** Effects of GSL-7 and GSN-4 on GSH-Px content in rat skin tissues. **(E)** Effects of GSL-7 and GSN-4 on MMP-1 content in rat skin tissues. **(F)** Effects of GSL-7 and GSN-4 on MMP-3 content in rat skin tissues. **(G)** Effects of GSL-7 and GSN-4 on IL-6 content in rat skin tissues. **(H)** Effects of GSL-7 and GSN-4 on IL-1β content in rat skin tissues. **(I)** Effects of GSL-7 and GSN-4 on TNF-α content in rat skin tissues. ^***^ *p* < 0.001, ^***^ *p* < 0.01, model group vs Control group; ***p < 0.001, GSL-7 group or GSN-4 vs model group, **p < 0.01, GSL-7 group or GSN-4 vs model group, *p < 0.05, GSL-7 group or GSN-4 group vs model group; **p < 0.01, GSL-7 group vs model group, *p < 0.05, GSN-4 group vs control group; *p ≤ 0.05, **p ≤ 0.01, ***p ≤ 0.001, ****p ≤ 0.0001 represents significant difference, # represents significant difference between model group and control group, * represents significant difference between model group and treatment group. Data are presented as *N* = 8. Abbreviations: GSL-7, total ginsenoside liposomes; GSN-4, total ginsenoside niosomes.

The schematic diagram of the rat skin photoaging modeling and repair process is shown in Figure 5A. An ELISA kit was used to determine the content of MDA in a skin tissue homogenate of rats in each group. MDA is the primary product of lipid peroxidation and can be used to measure the degree of skin anti-photoaging under oxidation. The results are shown in Figure 5B. Compared with the control group, MDA expression in the skin tissue of the model group was significantly increased (*p* < 0.01), indicating that UV radiation may cause skin lipid peroxidation and excessive MDA production. After treatment, the MDA expression was decreased in all the treatment groups (GS, GSL-7, and GSN-4). Furthermore, the decrease in the MDA expression was most pronounced in the GSL-7 group when compared with controls (*p* < 0.01). The expression of SOD and GSH-Px in the rat skin tissue homogenate was determined by using an ELISA kit. The expression of SOD and GSH-Px in the skin tissue also reflects the ability of the skin to resist oxidation and photoaging. The results are shown in Figures 5C, D. Compared with the control group, the activities of SOD and GSH-Px were significantly decreased in the model group (*p* < 0.001). The oxidative balance between the skin cells was also impaired. Compared with the model group, the SOD and GSH-Px expression was significantly increased in the GSN-4 (*p* < 0.05) and GSL-7 groups (*p* < 0.01). Therefore, it may be concluded that GS reduced UV radiation-induced skin-lipid peroxidation. GSL-7 and GSN-4 improved the transdermal efficiency and the percutaneous absorption of GS. According to the aforementioned experimental results, GSL-7 appears to have significant advantages over GSN-4 and a better therapeutic effect.

#### Effect of GSL-7 and GSN-4 on MMP Content

It has been shown that MMP-1 can degrade collagen Ⅰ and collagen Ⅲ. Moreover, MMP-3 can degrade collagen and elastin in a synergistic way, leading to collagen and elastin rupture (Lee et al., 2020; Wan et al., 2021). Therefore, MMP-1 and MMP-3 expression was measured in the rat skin tissue homogenate using ELISA kits (Figures 5E, F). Compared with the control group, MMP-1 and MMP-3 expression significantly increased in the model group. MMP-1 and MMP-3 expression decreased in all treatment groups, with the GSL-7 group showing the most prominent decrease (*p* < 0.01).

#### Effect of GSL and GSN on IL-6, IL-1β, and TNF-α Content

A cascade of reactions is triggered after UV exposure, with ROS triggering inducing the release of IL-6, IL-1β, and TNF-α (Zhou et al., 2021). ELISA kits were used to determine IL-6, IL-1β, and TNF-α expression in the rat skin tissue homogenate. The results are shown in Figures 5G–I. Compared with the control group, the expression of IL-1β, IL-6, and TNF-α was significantly increased in the model group (*p* < 0.001, *p* < 0.001, and *p* < 0.01, respectively). Compared with the model group, IL-6, IL-1β, and TNF-α were decreased in all the treatment groups, with the most marked decrease shown in the GSL-7 group.

In conclusion, GS can reduce the inflammatory factor expression in skin tissue following UV radiation. GSL-7 was more efficient than GSN-4 or GS in promoting GS absorption across the skin barrier.

#### Skin Appearance of Rats of Repair Photoaging

**FIGURE 6:**
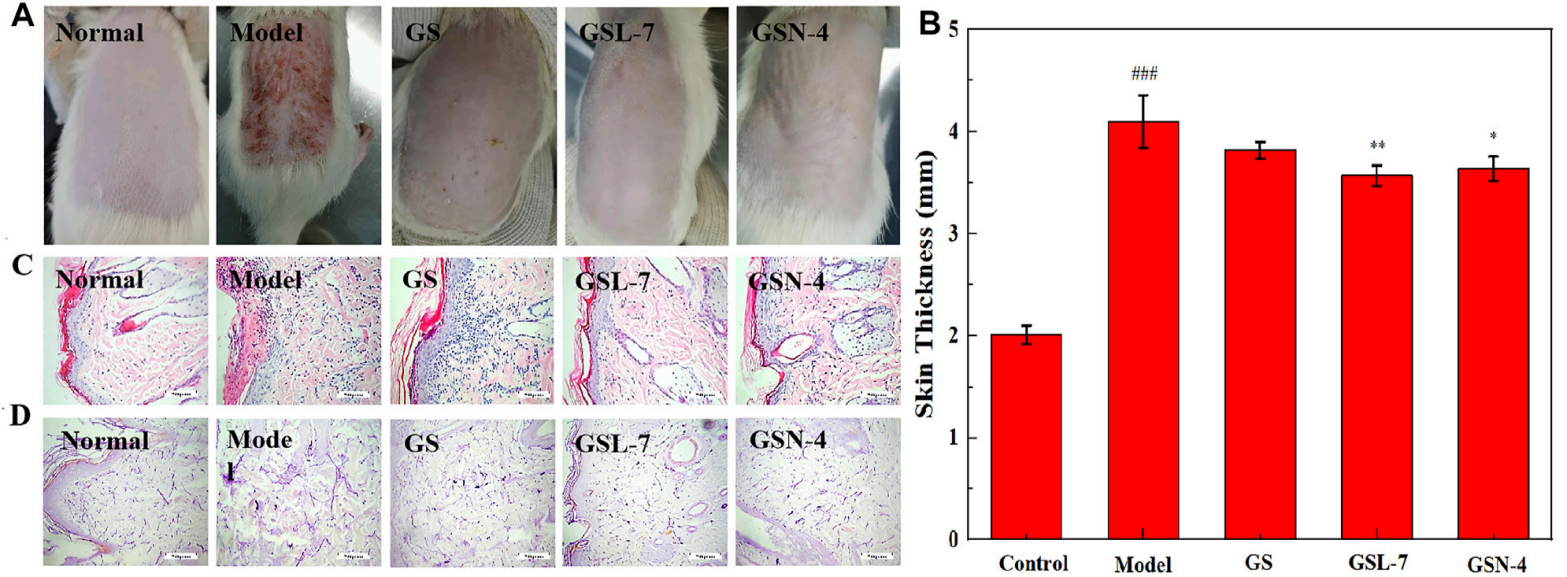
Skin appearance of rats of repair photoaging. **(A)** Skin appearance of rats after 10 days of administration; **(B)** Change of skin thickness, control group, **(C)** Histology of skin by H&E staining of the skin tissue section (×200); **(D)** Histology of skin by aldehyde fuchsine stain of the skin tissue section (×200). Data are presented as *N* = 8, **p < 0.01, GSL-7 group vs model group, *p < 0.05, GSN-4 group vs control group; ^###^ *p* < 0.001, model group vs control group; *p ≤ 0.05, **p ≤ 0.01, ***p ≤ 0.001, ****p ≤ 0.0001 represents significant difference, # represents significant difference between model group and control group, * represents significant difference between model group and treatment group. Abbreviations: GSL-7, total ginsenoside liposomes; GSN-4, total ginsenoside niosomes.

Clinically, photodamaged skin is characterized by the loss of elasticity, increased roughness and dryness, irregular pigmentation, and deep wrinkling (Bi et al., 2019). The results are presented in Figure 6. It can be seen from Figure 6A that the phenomena of rough skin, wrinkles, and abnormal thickness of the skin were all improved in the GS, GSL-7, and GSN-4 groups. The improvements were most pronounced in the GSL-7 group; the degree of rough skin was reduced and hair began to regenerate at the modeling site. Changes in the skin thickness of the rats in each group after treatment are shown in Figure 6B. Compared with the control group, the skin thickness in the model group was significantly increased (*p* < 0.001). On the contrary, compared with the model group, the skin thickness was decreased after the treatment, with the most marked effects observed in the GSL-7 group (*p* < 0.05). GS, GSL-7, and GSN-4 groups all received an acceptable therapeutic effect. GSL-7 had a therapeutic effect on photoaging.

Morphological changes in the rat skin were further analyzed by H&E staining and aldehyde fuchsin staining. The results are shown in Figures 6C, D. In the control group, the thickness of the epidermis was normal and the head of the epidermis and dermal papilla could be seen. In the model group, the stratum corneum was obviously thickened, the junction of epidermis and dermis was flattened, some epidermis in the damaged area was missing, and dermal papilla was rare or not present. After the treatment, signs of improvement were visible. In the GSL-7 group, the skin epidermis thickness was thin and close to normal, with a small amount of inflammatory cell infiltration at the junction of the epidermis and dermis.

Aldehyde fuchsin staining is predominantly used to visualize elastic fibers in the skin tissue and can be used to assess the elasticity of the skin. In the control group, the elastic fibers showed a relatively clear reticular structure with long and thin fibers, and an orderly arrangement and density (Figure 6D). In the model group, the arrangement of elastic fibers was visibly irregular. The reticular structure was not presented and there were many fractures, clusters, and accumulation. The treatment groups showed different degrees of improvement. The improvement was most evident in the GSL-7 group: accumulation and clustering had not occurred, and there was a clear network structure, with an orderly arrangement and consistent density.

## Conclusion

In this study, GSL and GSN were prepared and their skin anti-photoaging ability was assessed. The optimized GSL and GSN were characterized according to particle size, potential, EE%, drug transdermal release efficiency, and other parameters. The results showed that the particle size of GSL was smaller, more stable, and the EE% was higher than that of GSN. In the transdermal experiment, both GSL and GSN markedly improved the transdermal absorption efficiency of GS. The effect was more pronounced for GSL than the GS (48 h) and GSL-7 (48 h) groups, with a 48-fold total skin transmittance. In addition, equal doses of GS, GSN, and GSL showed that GSN and GSL effectively inhibited lipid peroxidation, and increased the SOD and GSH-Px expression in HaCaT cells. GSL and GSN also increased HaCaT cell proliferation, migration, and uptake *in vitro*. The expression of MMPs and inflammatory factors in the skin tissue was reduced by GSN and GSL, indicating that GS treatment—most notably with GSL—improved the ability of the skin to repair UV-induced skin tissue damage. In conclusion, GSN and GSL can be used as transdermal drug delivery vehicles of GS to enhance the anti-photoaging ability of GS in the skin. GSL may have significant potential as an effective treatment for skin photoaging, and further studies into its anti-inflammatory effects are required.

## Data Availability

The original contributions presented in the study are included in the article/Supplementary Material, further inquiries can be directed to the corresponding authors.
